# Side- and similarity-biases during confidence conformity

**DOI:** 10.1371/journal.pone.0253577

**Published:** 2021-07-16

**Authors:** Winny W. Y. Yue, Kiyofumi Miyoshi, Wendy W. S. Yue

**Affiliations:** 1 Department of Psychology, The University of Hong Kong, Pok Fu Lam, Hong Kong; 2 Department of Psychology, University of California Los Angeles, Los Angeles, California, United States of America; 3 Department of Physiology, University of California San Francisco, San Francisco, California, United States of America; University of Padova, ITALY

## Abstract

Memory conformity may develop when people are confronted with distinct memories reported by others in social situations and knowingly/unknowingly adhere to these exogenous memories. Earlier research on memory conformity suggests that (1) subjects were more likely to conform to confederate with high confidence; (2) subjects with low confidence on their memory accuracy were more likely to conform, and; (3) this subjective confidence could be adjusted by social manipulations. Nonetheless, it remains unclear how the confidence levels of ours and others may interact and produce a combined effect on our degree of conformity. More importantly, is memory conformity, defined by a complete adoption of the opposite side, the result of a gradual accumulation of subtler changes at the confidence level, i.e., a buildup of confidence conformity? Here, we followed participant’s confidence transformation quantitatively over three confederate sessions in a memory test. After studying a set of human motion videos, participants had to answer simultaneously whether a target or lure video had appeared before by indicating their side (i.e., Yes/No) and their associated confidence rating. Participants were allowed to adjust their responses as they were being shown randomly-generated confederates’ answers and confidence values. Results show that participants indeed demonstrated confidence conformity. Interestingly, they tended to become committed to their side early on and gain confidence gradually over subsequent sessions. This polarizing behaviour may be explained by two kinds of preferences: (1) Participant’s confidence enhancement towards same-sided confederates was greater in magnitude compared to the decrement towards an opposite-sided confederate; and (2) Participants had the most effective confidence boost when the same-sided confederates shared similar, but not considerably different, confidence level to theirs. In other words, humans exhibit side- and similarity-biases during confidence conformity.

## Introduction

Quick dissemination of information via new media has enhanced shared exposure to a variety of information or point of view. However, people’s sets of memories towards the same event are often not identical due to individual differences in perception [1] and the level of memory loss/corruption each experienced [2–4]. When we encounter these nonidentical sets of memories, we may knowingly or unknowingly be influenced due to social pressure and/or our desire to be accurate, thus reporting to adopt memories congruent to other’s [5, 6]. This process is called “memory conformity”.

In laboratory settings, memory conformity is usually tested by including social manipulation (i.e., engaging participants with confederate(s)) in a two alternative forced choice (2-AFC) memory test. A participant is considered to have conformed if he/she abandons his/her original choice and report the alternative upheld by the confederate, i.e., demonstrates a complete side-shift. Once people have conformed, they may retain the conformed memories for as long as 7 days, even when social influences have been removed [7]. If these conformed memories are inaccurate, such lasting effects may come with alarming outcomes: it may trigger stress [8] and/or modify our behaviours [9]. The influence of others on our behavioural reaction may vary based on our subjective confidence on our memory accuracy. For example, we may use different memorization strategies depending on our confidence on our memory; specifically, under-confident people tend to set more external reminders [10].

People’s subjective confidence in their memory accuracy is found to be adjustable in response to confederate’s answers. Specifically, their confidence decreased after being exposed to multiple confederates during social manipulation, no matter they have conformed or not [7]. However, researchers have only examined participants’ confidence change in a single confederate session. They largely overlooked whether and how these confidence change may pile up over multiple confederate sessions and lead to side shifting (i.e., memory conformity as defined above).

What are some confederates’ characteristics that would encourage people to change their confidence and/or conform? Many studies have investigated features that are related to how people perceive other’s credibility, e.g., age, expertise, time spent on memorizing the information, etc. [4, 11]. Nevertheless, these features are largely inaccessible in reality when we meet with strangers, start a spontaneous conversation, or read people’s comments online. Instead, during the above circumstances, other’s tone, facial expression, choice of words, etc., are usually more readily available. Some of these features, such as pitch, speech rate, are thought to be associated with one’s confidence [12, 13]. In other words, confederates may still be able to convey confidence information to us even when only language features are prominent. So far, only few studies have tried to link a higher confederate confidence to higher conformity tendency. Goodwin et al. (2017) manipulated confederate’s confidence with scripted statements during a discussion where participants were required to recall an event collaboratively with the confederate [14]. However, the way the experiment was designed and executed may carry some limitations: (1) participant’s pre-confederate responses were not collected, thus it was not possible to determine whether conformity had indeed occurred; (2) the scripted expressions that the confederates used (e.g., “maybe”, “I am not sure”) might have led to ambiguous interpretation; (3) in a face-to-face discussion, confederates’ vocal-gestural delivery might not be uniform. A second study presented confederate’s confidence by a 10-point scale during social manipulation and is the only study in the field using quantified confidence values. Following a highly confident confederate, participants were found to develop higher confidence [15]. It is worth pointing out that their confidence change (i.e., the pre- and post-confederate differences) was not captured as researchers use separate item sets to collect participants’ pre-confederate and post-confederate confidence ratings for comparison.

### Confidence conformity and current study

The above evidence, although meager in various ways, suggests that our conforming tendency is affected by the confidence of the confederates; we are also more likely to conform when we have low confidence ourselves [16]. The immediate question is: how do these two sets of confidence interact and collectively determine the degree we would conform? If exposing to confederates could in turn modify our subjective confidence as suggested above, how would our confidence evolve after multiple confederate sessions? Would the effect of confidence conformity (i.e., the tendency to follow other’s reported confidence) be accumulative, leading gradually to side shifting?

In the current experiment, we study how our confidence on the accuracy of our memory may be affected by other’s confidence and how a possible bias in such confidence adjustment could gradually enhance people’s confidence over time, thus leading to confidence polarization. We tracked participants’ changes in response and confidence in a memory test with three confederate sessions where confederates’ response and confidence level would be shown in the form of numbers in each session. Instead of using static visual scenes, we focused on visual human motion memory that was formed from watching videos and was later retrieved in a simulated online environment as we recognized its importance in a multitude of daily activities, including skill acquisitions, non-verbal communication [17, 18], and its assistant role in our recall of other types of memory [19]. In addition, convenient social media transmission has made videos involving human motion increasingly universally accessible and even more impactful.

## Results

**Fig 1:**
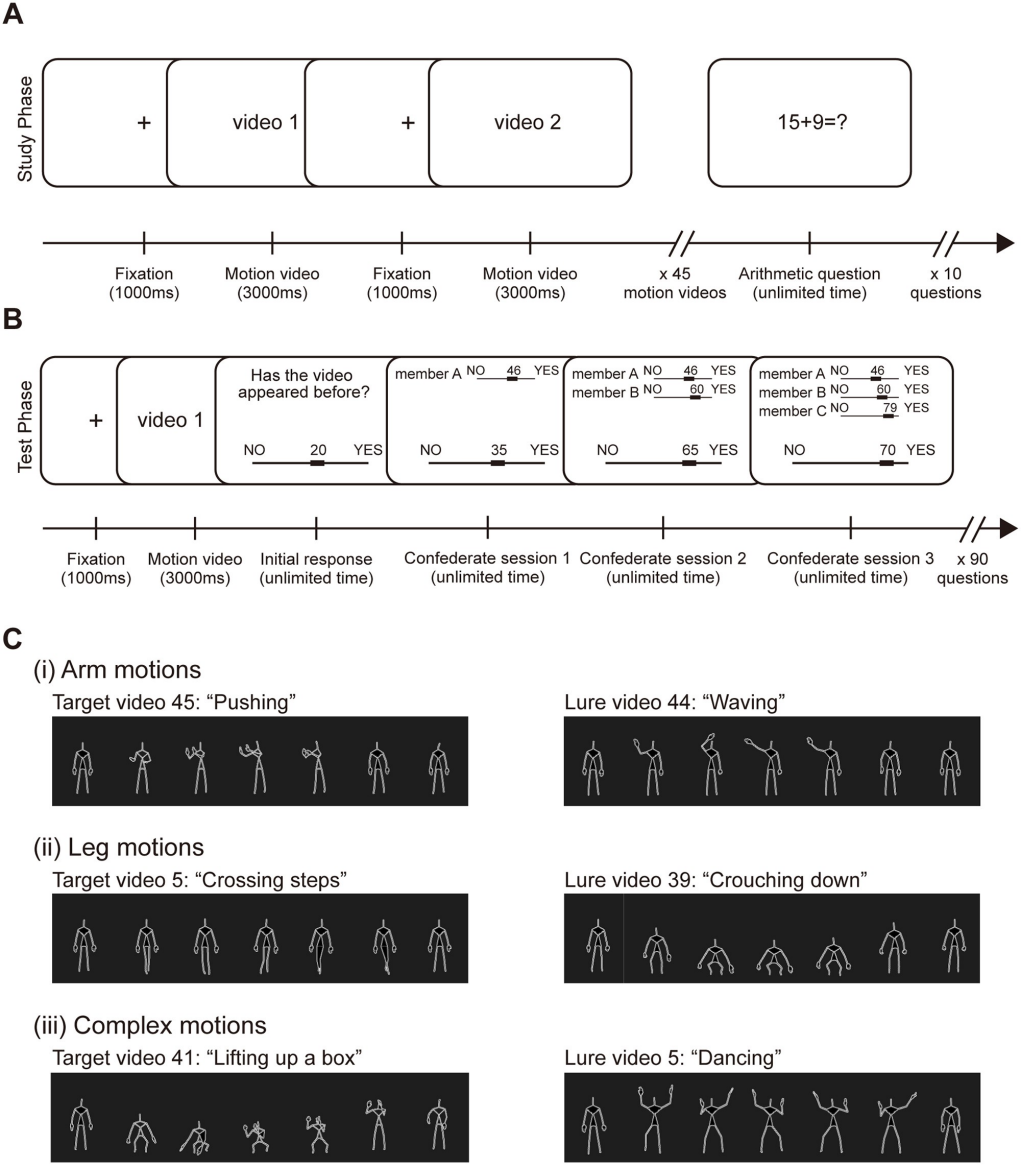
Experimental design. (A) Study phase of the experiment. Participants were required to memorize a total of 45 three-second skeletal motion videos appearing in random orders, followed by answering 10 arithmetic questions to prevent memory rehearsal. (B) Test phase of the experiment, with altogether 90 questions (45 target and 45 lure videos). Participants were asked to indicate with a slider their answers and confidence to the question “Has the video appeared before?”. After the initial response, participants entered three consecutive confederate sessions. In each session, one additional computer-generated confederate confidence rating would be shown and participants would have a chance to change their slider value with the goal of getting the highest overall group score. (C) Examples of computer-rendered motion videos. The target and lure videos contained equal number of arm, leg and complex motions (see also S1 Table).

In this study, we follow how people may adjust their subjective confidence on their memory accuracy and conform to the opinions of others when engaged in some visual human motion memory tasks in a simulated online environment. Thirty-eight participants were recruited to the following two-phase memory test: in the study phase, participants were required to memorize 45 three-second skeletal animations (the targets) appearing in a random sequence (Fig 1A, See Methods for full details). These videos were generated from video-recorded motions performed by an actor and were computer-rendered to minimize distractive features, such as human faces, clothes, etc. The motions included movements of different body parts, involving arms, legs or more complex combinations of the above; a substantial percentage of these are commonly recognizable bodily movements, such as clapping, running and crawling (Fig 1C; see also S1 Table).

The study phase was followed by a test phase consisting of 90 randomly-appeared questions (45 target and 45 lure motion videos, see S1 Table), in which participants had to report how confident they were to the question of whether the motion video shown had appeared before. Participants indicated their answers by using a virtual slider ranging from −100 (NO) to +100 (YES) with 0 representing a neutral decision; these answers are deemed initial responses (R_0_). We employed this slider answering scheme in order to collect participants’ answers and confidence ratings in a single step, so as to avoid post-decision confirmation effect if participants were asked more than once [20]. To better control the task difficulties, we intentionally adjusted the number and distinctiveness of motions included (see Materials and methods). Motions that involve similar gesture or perceived meaning but with different intensities, for example, small jump and big jump, cleaning window and slapping, were separately assigned as targets and lures (S1 Table, column 6). S1 Fig shows that the mean initial response across all participants did not go beyond ±50 for all videos tested, suggesting a reasonable level of difficulty. The mean initial response also spanned a range, with a few even crossing to the “incorrect” side, thus allowing us to investigate how conformity might be correlated with variations in initial response.

To investigate how one’s confidence is influenced by others, we designed three confederate sessions at the end of each initial response and captured participant’s confidence transformation (Fig 1B). In each confederate session, a participant would be presented with a confederate’s confidence value (in the form of a slider and a number display) randomly generated within a certain range defined by the participant’s initial response (see Materials and methods and S2 Table). Participants had to indicate their response again in each session, thus giving a total of one initial response (R_0_) and three confederate session responses (responses R_1_, R_2_ and R_3_ for sessions with C_1_, C_2_ and C_3_, respectively). Henceforth, we shall call the participant’s confidence rating before and after viewing a certain confederate value––R_pre_ and R_post_––respectively, regardless of session number whenever suitable.

We confirmed that participants did consider the confederate values by computing the Pearson correlation coefficients between the before-and-after difference in responses (R_post_ − R_pre_) and the confederate-participant difference (C − R_pre_) across videos (S2 Fig). Strong positive correlation exists only for the current but not past videos (blue diagonals) in all sessions, suggesting that participants did respond to the confederates’ ratings and, more importantly, did not form from experience either a significantly positive or negative impression of whether a particular confederate is trustworthy or not. Therefore, each video could be analyzed independently.

### Dissection of side shifting behaviour into different types of conforming behaviours

**Fig 2:**
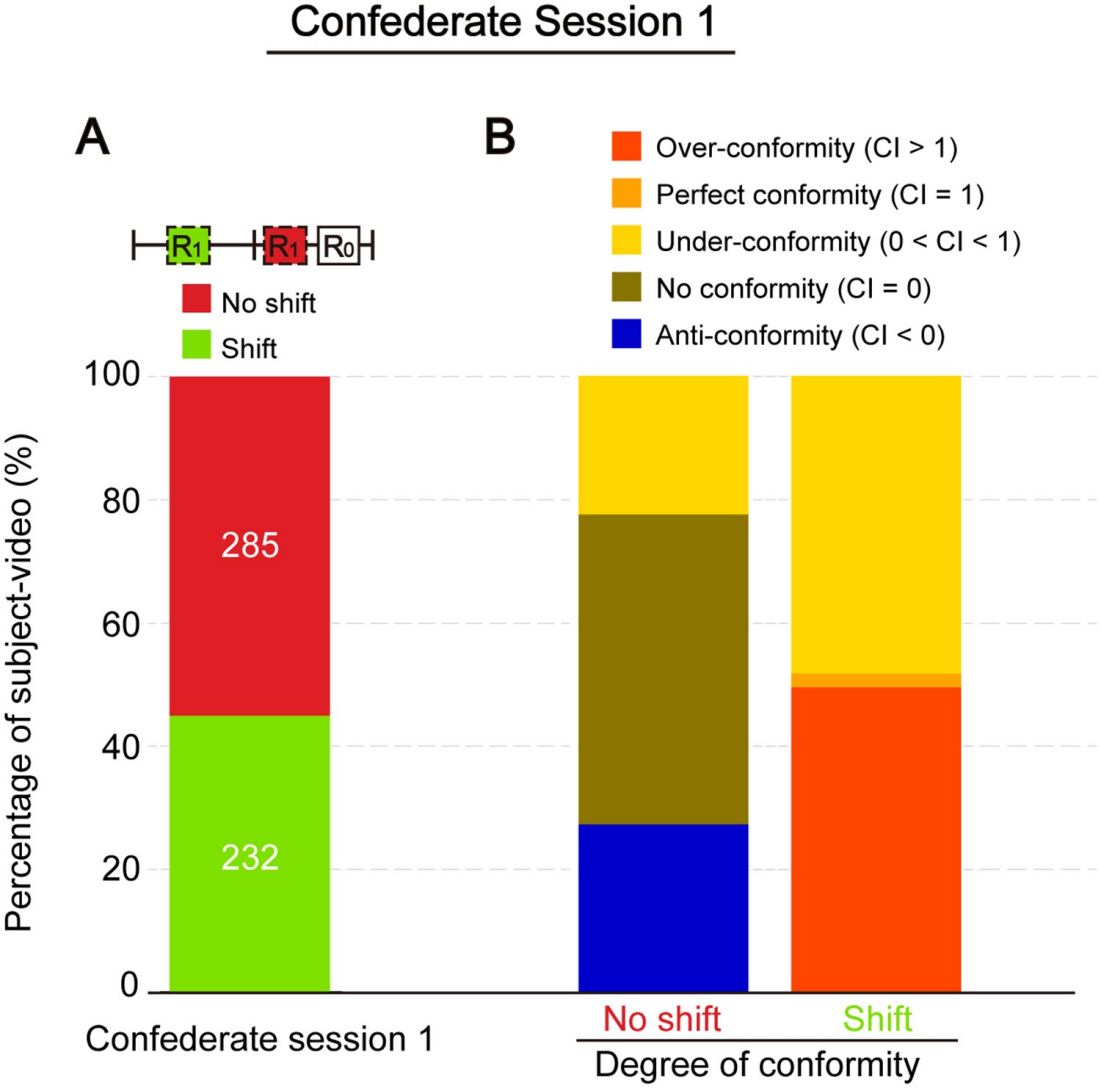
Different conforming behaviours to an opposite-sided confederate in session 1. (A) Percentage of subject-videos who shifted and did not shift side after being shown the first confederate value (C_1_) that was on the opposite side of their initial response (R_0_). Number of datapoints is indicated on the bars (n = 517). (B) The no-shift and shift group in (A) further classified by the degree of conformity based on participants’ conformity index (CI).

In past studies, memory conformity was usually examined by calculating the proportion of participants who complied to an opposite standpoint [4]. We began by adopting this conventional definition and looked at how participants would adjust their response (R_1_) in the first confederate session by focusing on side-shifting. Subject-video trial with C_1_ on the opposite side of R_0_ were isolated (n = 517), and the percentages of participant who had shifted and not shifted side are presented in Fig 2A. Nearly half of all participants (44.8%) in this group had shifted side in the first session. Among these, around 97% of participants had a R_0_ ≤ 30, consistent with the result of a previous study that participants with low confidence have a high conforming tendency [16]. This high conforming rate may also be partly due to a heightened degree of anonymity in the current computer task, which might have reduced participant’s self-awareness to maintain a persistent belief, compared to face-to-face confederate’s responses [21].

In order to measure the degree of conformity more quantitatively, we calculated the conformity index (CI) by taking the ratio between the change in response (R_post_–R_pre_) and the corresponding confederate-vs-self difference (C–R_pre_), that is:

$$\text{Conformity}\;\text{Index}\;(\text{CI})=\frac{{\text{R}}_{\text{post}}-{\text{R}}_{\text{pre}}}{{\text{C}-\text{R}}_{\text{pre}}}$$ (1)

The introduction of CI allowed more direct comparisons across trials and sessions with different R_pre_ and C, the two parameters which we had little to no control on due to experimental settings (see Materials and methods).

The CI conveniently describes different types of conforming behaviours, including: (1) anti-conformity when a participant’s confidence increase or decrease in response to a smaller or larger C, respectively (CI < 0); (2) no conformity when a participant did not change their response (CI = 0); (3) under-conformity when a participant shifted mildly towards but did not completely adhere to C (0 < CI < 1); (4) perfect conformity when a participant followed exactly the value of C (CI = 1), and; (5) over-conformity when a participant followed C and shifted beyond it (CI > 1).

By using CI, we could decompose the shift and no shift groups in confederate session 1 into different conformity types, indicating that conformity can be regarded not as an all-or-none process (Fig 2B). The shift group is made up of an almost equal proportion of under-conforming and over-conforming participants. In contrast, under-conformity constitutes a much smaller percentage of the no shift group; the majority in this group did not change their responses at all. Unexpectedly, some of those who maintained their side even increased their initial confidence in response to the opposite confederate rating. These participants had a relatively strong belief (shift group: mean of R_0_ ± SD = 15.039 ± 11.505, n = 232; no shift group: mean of R_0_ ± SD = 35.867 ± 32.974, n = 285; p < 0.001, with t-test) and hence a possible tendency to interpret an opposite-sided answer as an inaccurate, thus untrustworthy, response.

### Similarity effect during conformity

**Fig 3:**
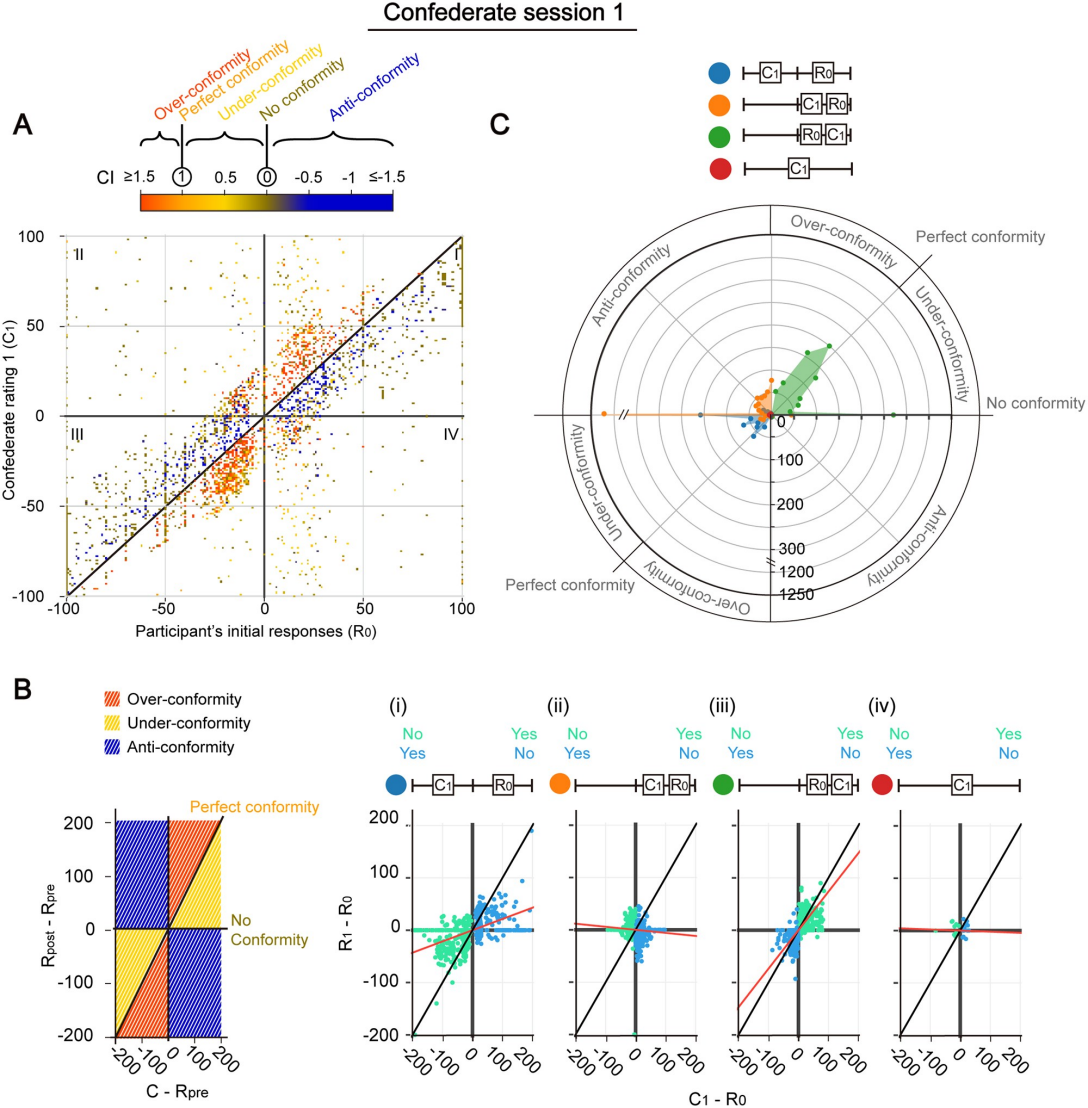
Conforming behaviours with respect to R_0_ and confederate confidence in session 1. (A) Plot of confederate value (C_1_) against participant’s initial responses (R_0_). Each dot represents a subject-video, with colour representing the conformity index (CI). Black diagonal line is R_0_ = C_1_. n = 3345. Data points with invalid CI (e.g., when R_0_ = C_1_) were excluded. (B) Plot of change in response (R_1_ − R_0_) against the difference between C_1_ and R_0_. Left panel, schematic diagram depicting the different conformity types (coloured as in Fig 3A) mapped onto each zones and lines. Right panels, actual data, grouped based on the relationship between R_0_ and C_1_: column 1, C_1_ being on the opposite side of R_0_; columns 2, C_1_ remains on the same side but being less or equally confident to R_0_; columns 3, C_1_ remains on the same side but being more confident than R_0_; column 4, C_1_ = 0. Data in green are with R_0_ ≥ 0 whereas data in blue are with R_0_ < 0, also schematized by a flipped slider in the symbols above. Red lines are the best fit lines, with the slope reflecting the average CI, of all the points on the graph. R_0_ = 0 and datapoints with invalid CI were excluded, n = 3360. (C) Radial histogram showing the number of participant-videos with different ranges of CI. Quadrants are arranged as in (B), with the types of conformity behaviour reiterated on the outer circle. Participant-video counts (see labelled axis) were obtained by mapping the data points in (B) on a polar coordinate and by binning together data points that fell within 10-degree angular intervals to more clearly illustrate the distribution. Data with R_0_ ≥ 0 [green points in (B)] and R_0_ < 0 [blue points in (B)] were combined by reflecting the latter over origin (i.e., flipping ratings over to the opposite side on the slide bar).

A few studies have suggested that people’s likelihood to conform to other’s memory is associated separately with their internal confidence and the confidence level of the confederates [4, 6, 15, 16]. However, it is unclear how these two qualities may interact during the development of conformity. Hence, we looked into how CI may vary with the levels of self and confederate confidence in the first confederate session of our memory test. Fig 3A shows a heat-map of C_1_ against R_0_ with the colour of each datapoint representing the CI of a single subject-video event. The diagonal marks the special case of R_0_ = C_1_ and a notable divide in CI: in quadrant I (i.e., when both R_0_ and C_1_ are on the “Yes” side of the answer slider), CI is mostly positive above whereas negative below the diagonal. This sudden flip in sign is rooted in the definition of CI (with C_1_ –R_0_ being the denominator). An anti-conformity here below the diagonal implies that participants did not follow a less-confident confederate but had instead raised their confidence. Despite this sign difference, the magnitude of CI is consistently high on both sides of the diagonal but becomes less extreme as one moves away from the diagonal. Thus, confidence enhancement per unit difference in confederate-vs-self confidence is stronger when the participant encounters a similarly confident confederate. Altogether, these observations suggest that participants would reference their internal confidence level when deciding how much to conform.

Curiously, a clear positive/negative divide in CI is not observed in quadrant IV (i.e., when R_0_ is on the “Yes” and C_1_ on the “No” side of the answer slider). Conformity appears uniformly weak in this space, although datapoints are scarce due to the constraints we applied on the generation of confederate ratings (see above and Materials and methods). Over-conformity and anti-conformity do occur occasionally, especially around the origin, but they appear randomly mixed and show no obviously identifiable pattern. All in all, the conforming pattern appears different toward same-sided (quadrant I) versus opposite-sided (quadrant IV) confederates, reflecting a side bias against the latter. Similar observations can be derived from quadrants II and III (with R_0_ on the “No” side of the answer slider), which show almost the same general conforming patterns as quadrants I and IV but reflected through the origin.

To more directly illustrate how participants would adjust their response according to the confidence gap between confederate and self, we plotted (R_1_ –R_0_) against (C_1_ –R_0_) in Fig 3B. Under this setting, the CI would be represented by the slope of an individual datapoint to the origin (averages as red lines in Fig 3B). In other words, depending on the associated conforming behaviour, the datapoint would situate in different zones on the x-y plane (coloured in Fig 3B, left). For easier comparison, we separated datapoints into 4 groups based on the major types of situation-specific conforming tendencies identified in Fig 3A, namely: (1) when C_1_ is on the opposite side of R_0_, (2) when C_1_ is on the same side but less confident than R_0_, (3) when C_1_ is on the same side and more confident than R_0_, and (4) when C_1_ equals to zero. Fig 3B largely recapitulates Fig 3A in showing a bias for conforming to confederates holding the same belief in comparison to opposite-sided confederates (Fig 3Bii and 3Biii vs Fig 3Bi). In addition to these general trends, it can also be appreciated from Fig 3B that participants demonstrate more consistent confidence change in some situations than in others; for example, datapoints appear to cluster more tightly in the case of a same-sided and more confident confederate (Fig 3Biii), compared to that of a same-sided and less confident confederate (Fig 3Bii).

This difference in the distribution of CI can be more clearly visualized with collective data in the radial histogram in Fig 3C. Here, we transformed the Cartesian coordinates of each point in Fig 3B into polar coordinates and binned together datapoints that lied within a 10-degree angular interval. Thus, the slope of each point to the origin in Fig 3C still reflects the CI while the height of the point displays the number of participants choosing that CI interval. Given that the datapoints are similarly distributed in the R_0_ > 0 and R_0_ < 0 quadrants in Fig 3B, we reflected the R_0_ < 0 datapoints over the origin and lumped them with the R_0_ > 0 datapoints to obtain a single distribution profile for each group for simpler comparisons. All three groups show a significant number of zero CI. However, it can be clearly seen that the three groups manifest distinct CI distributions: the group with same-sided, more confident confederates (green) gives a sharp peak at weak over-conformity whereas the group with same-sided, less confident confederates (orange) has a more even distribution throughout the anti-conformity zone, again consistent with the finding above. Opposite-sided confederates (blue) also produce a fairly even distribution, but largely confined within the under-conformity zone. Clearly, participants expressed certain biases in their conformity response.

**Fig 4:**
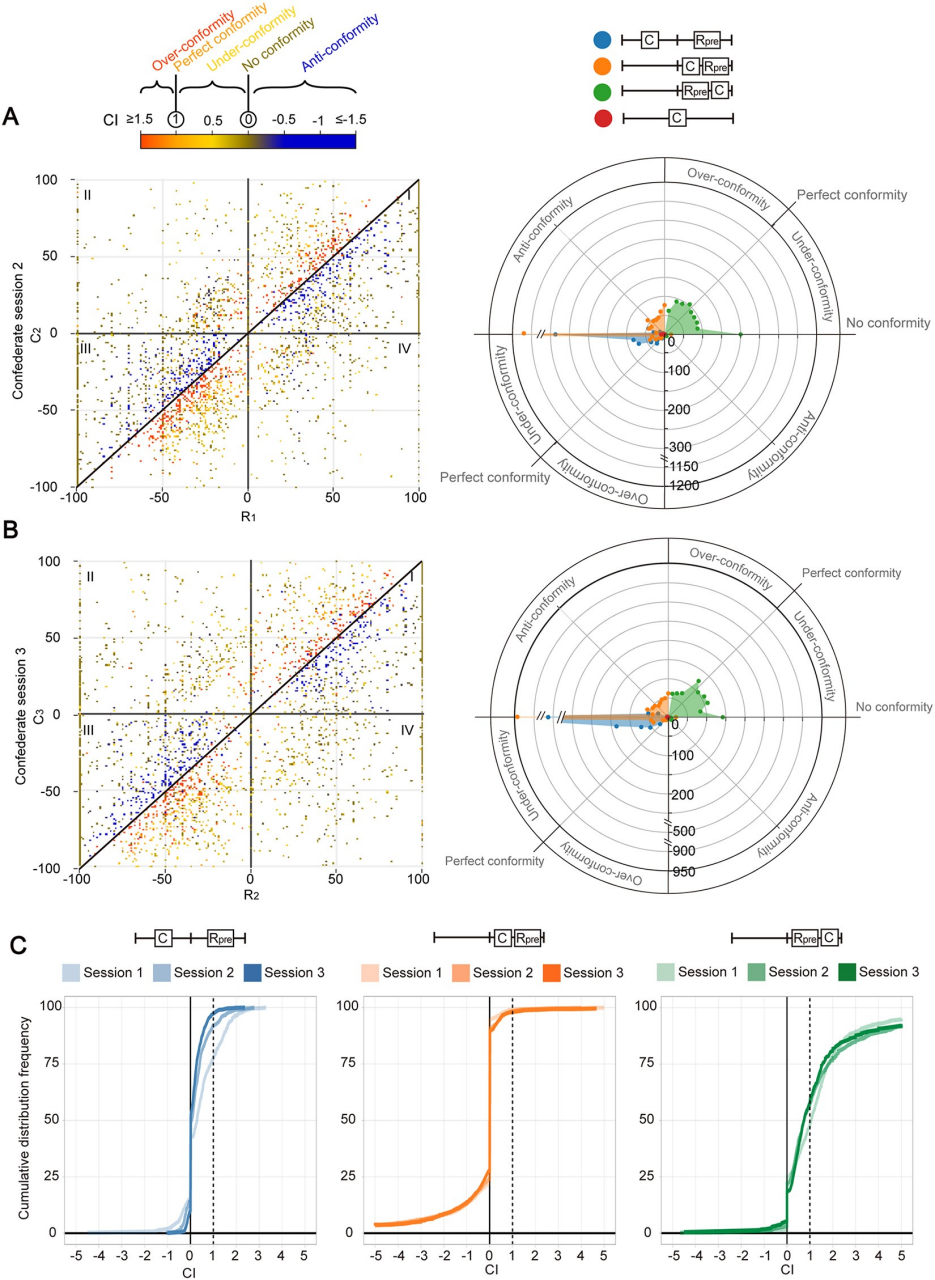
Level of conformity to the second and third confederate’s answers. (A and B) Same as Fig 3A and 3C but for session 2 (panel A, n = 3377) and session 3 (panel B, 3376). (C) Cumulative distribution frequency plots showing the distribution of CI in the three sessions. CI calculated and colours indicated as in Fig 4A and 4B (Right). Dotted lines are plots CI = 1. Data points with invalid CI were excluded. Left: n = 521, 647, 899 for session 1, 2, 3; Middle: n = 1665, 1731, 1497 for session 1, 2, 3; Right: n = 1079, 940, 916 for session 1, 2, 3.

Remarkably, participants’ behaviours in confederate sessions 2 and 3 (Fig 4) grossly resemble those in session 1. More specifically, (1) a side bias becomes even more apparent as datapoints with opposite-sided C start to populate in later confederate sessions due to the gradual shift in constraint we imposed on the generation of C (see above and Materials and methods), and (2) there is again a prominent confidence bias towards confederates with similar confidence to the participants’ (as reflected by the more extreme CI just above and below the diagonal in Fig 4A and 4B, left). Nonetheless, there are a number of notable differences across confederate sessions. First, the number of non-conforming participants in the groups with same-sided confederates (green and orange) decreases over sessions. Moreover, a more even CI distribution is seen in the group encountering a confederate holding the same and stronger belief (green). Furthermore, there are fewer over-conforming participants in the group with an opposite-sided confederate (blue). To capture this shift in behaviour over sessions, we overlaid the cumulative distribution frequency of CI of the three sessions for each of the three groups (Fig 4C). Kolmogorov–Smirnov tests suggest that there are significant differences between confederate sessions 1 and 3 in all three groups (group 1, blue, p < 0.001; group 2, orange, p < 0.05; group 3, green, p < 0.001), most likely attributed to one or more of the differences mentioned above.

### Transformation of participant’s confidence in response to confederate across sessions

**Fig 5:**
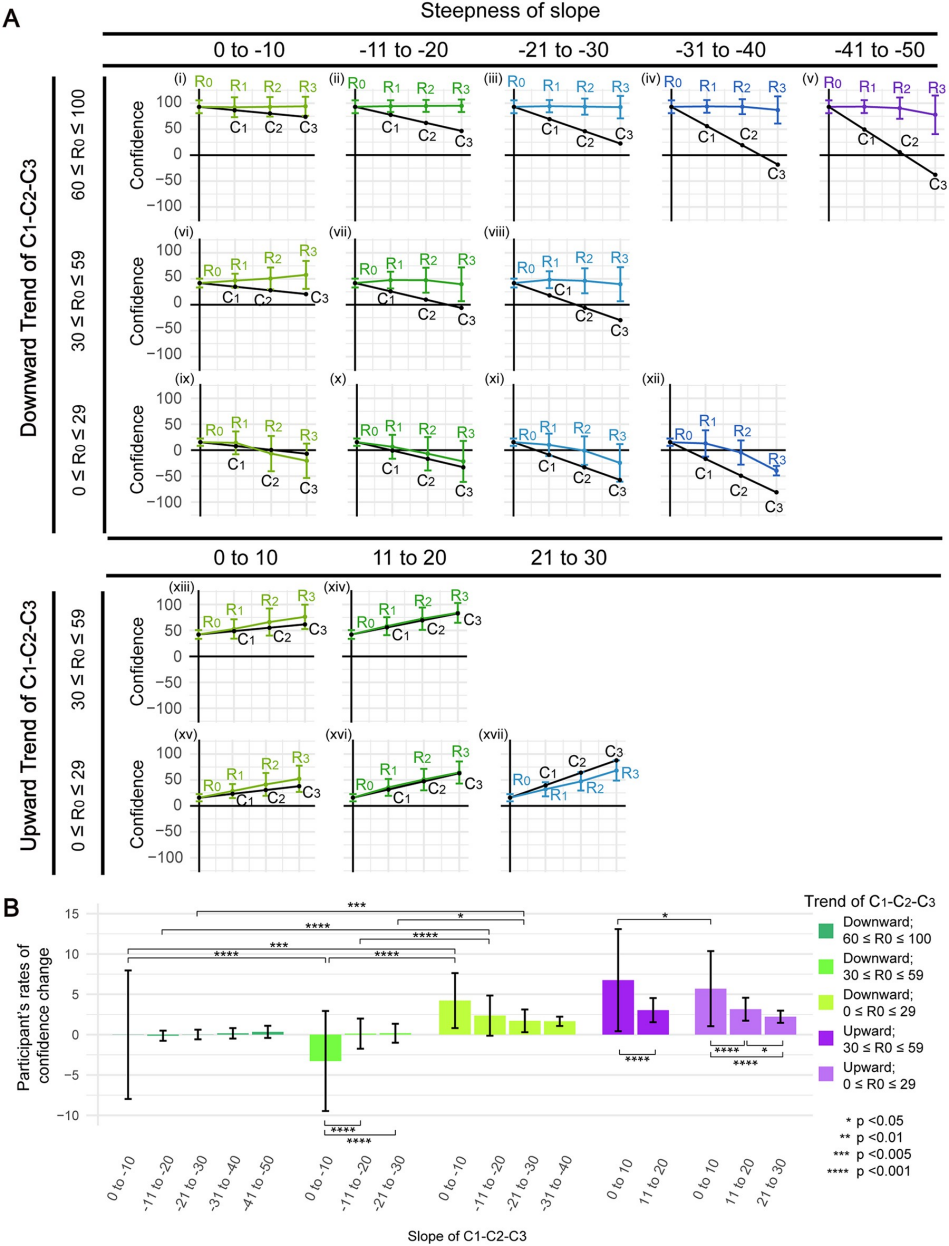
The effect of increasing/decreasing confederates’ confidence on participants’ responses. (A) Participants’ responses (colour lines, mean ± SD) across sessions with different levels of initial confidence and trends of confederate values. Participants are further grouped based on their average slope of C_1_- C_2_ and C_2_- C_3_, represented by the black lines. Data with R_0_ < 0 were flipped in the three sessions as in Fig 3C. (B) Participants’ rates of confidence change (mean ± SD) in the three confederate sessions (see Materials and methods). Significantly differences (pair-wise t test) are marked by stars. From left to right, n = 173, 281, 86, 162, 130, 79, 175, 38, 7, 312, 98, 2, 103, 84, 126, 379, 104.

The above analyses focused only on individual sessions. Next, we ask how these single-session behaviours may add up over multiple confederate sessions. In particular, how would participants react to several increasingly affirmative or disaccordant confederates? In Fig 5, we isolated subject-video sessions that have either an upward (C_1_ < C_2_ < C_3_) or downward (C_1_ > C_2_ > C_3_) confederate confidence trend (mean as black lines in Fig 5) and grouped them by the steepness of the trend (columns in Fig 5). These groups were further divided into sub-groups of low, medium and high R_0_ (rows in Fig 5) as we expect from previous studies and our above data that people’s subjective confidence may affect their degree of conformity. Presented on the plots as coloured lines are the mean ± SD of participants’ responses across sessions (i.e., R_0_, R_1_, R_2_ and R_3_); for simplicity, data are reflected over origin as in Fig 3C.

Intriguingly, for groups that saw a downward C_1_-C_2_-C_3_ trend (Fig 5Ai–5Axii), a steeper trend did not produce a significantly greater effect on participants’ response (quantified in Fig 5B, see legend). Participants with medium to high R_0_ almost always maintained or sometimes even increase (Fig 5Avi) their confidence over the three confederate sessions, showing at most a mild but insignificant dip in confidence when encountering an opposite-sided confederate in the last session (Fig 5Av). Side-shifting seems to occur only to participants with low R_0_, who appear to be more substantially influenced, but even in this case, a steeper downward C_1_- C_2_- C_3_ trend is not necessarily more effective in changing participant’s confidence (Fig 5B). In stark contrast, for groups that saw an upward C_1_-C_2_-C_3_ trend (Figs 5Axiii–5Axvii), the steepness of the trend does matter: a gentler slope is actually more effective in eliciting a confidence increase. This is perhaps contrary to what most may have expected but is consistent with one’s preference on similarly confident confederates as seen in individual sessions above. Participants’ R_0_ seems to have a lesser impact here.

### Confidence polarization

**Fig 6:**
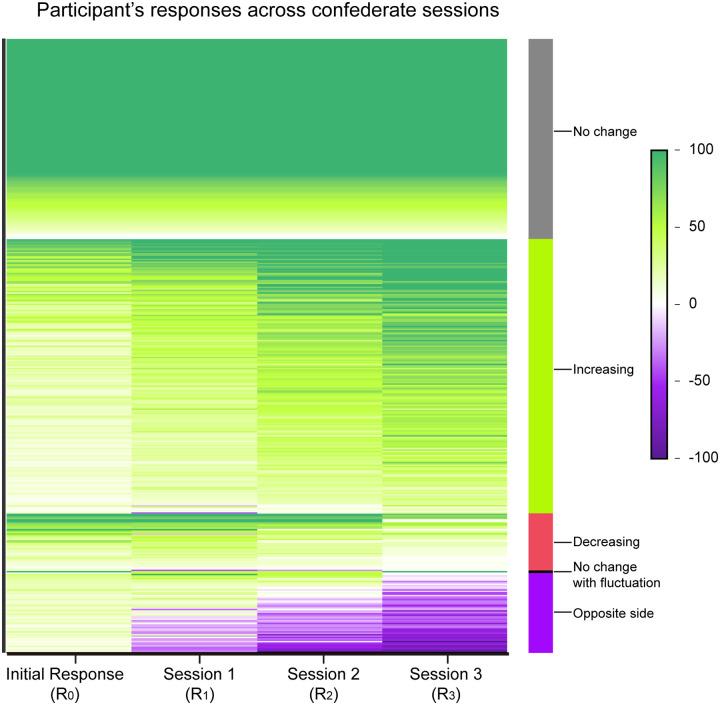
Participant’s responses across sessions. Heat-map plot of participants’ confidence ratings across sessions. Each line shows the responses of a participant to a particular video (n = 38 participants x 90 questions = 3420 lines). Lines are grouped and ordered by the response trend (i.e., no change, increasing, decreasing, etc.) as marked by the colour bars immediately on the right. When R_0_ < 0, all participant’s responses and subsequent confederate’s confidence ratings in that particular trial were flipped as in Fig 3C.

A stronger positive responsivity toward same-sided rather than opposite-sided confederates may provide a driving force for confidence polarization. To gain a global view on how participants’ confidence evolves across session, we plotted all participants’ responses across sessions for all 90 video tests in the heat-map in Fig 6, ordered by similarity in response profiles after data reflection as above. Two predominant types of behaviour were found: First, participants who had a relatively high initial confidence stayed firm across confederate sessions (i.e., R_0_ = R_1_ = R_2_ = R_3_). Second, the majority of participants who started off with low initial confidence increased their confidence, usually gradually, over the three confederate sessions (i.e., R_3_ > R_0_). We also observed a small group (~9%) of participants, who experienced a confidence reduction (i.e., R_3_ < R_0_) but still stayed on the same side. Complete side-shifting (i.e., R_3_ being on the opposite side as R_0_) is uncommon, accounting only for ~13% of all sessions. Among these cases, there is frequently also a gradual increase in confidence after a participant committed to the opposite side. The net result of all the above behaviours is a more extreme pattern of confidence levels in the last response or in other words, confidence polarization. Part of this polarization may be explained by the increasing/decreasing trend in confederate values across sessions that we intentionally skewed toward when we set up the experiment (as in Fig 5), but in a considerable number of cases, we did not actually see such a corresponding trend (S5 Fig). This suggests that polarization does not arise purely from acquisition of biased information, but quite possibly, also from psychological preferences towards specific side and similar confidence of confederates as identified in the above sections.

### Can we still change when our confidence is high?

**Fig 7:**
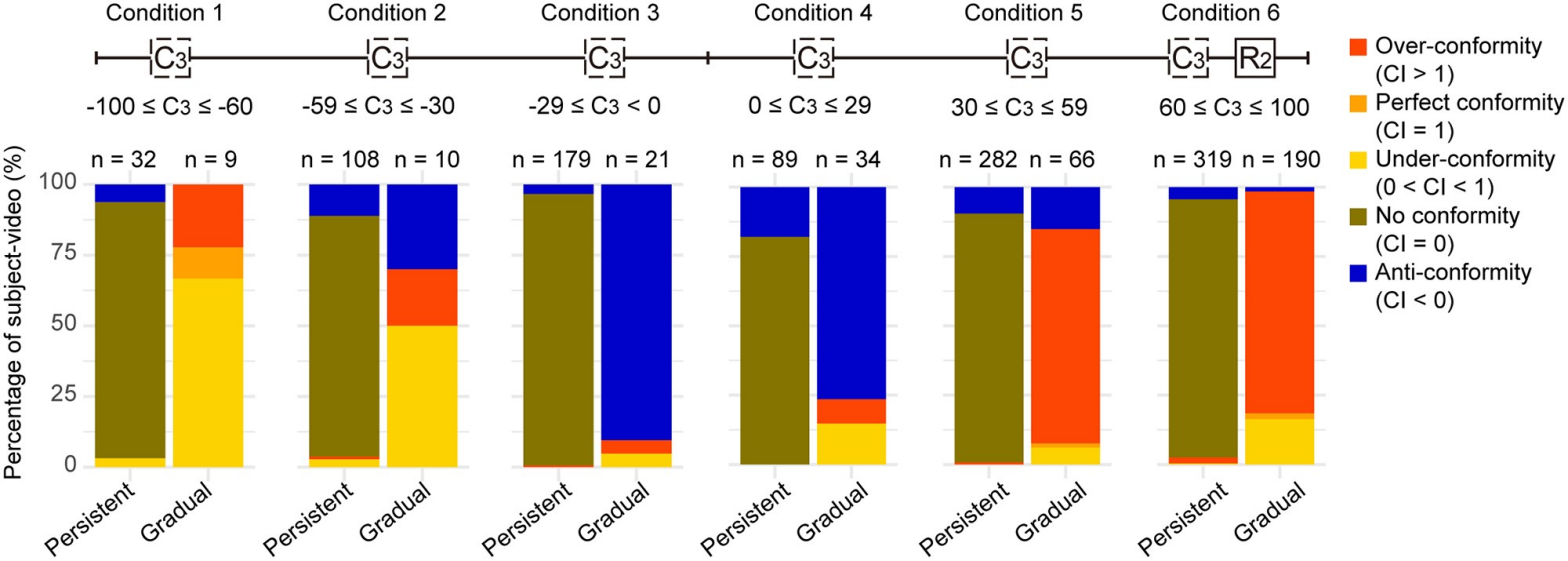
Conforming bebaviours of highly-confident participants in confederate session 3. The percentage of conforming behaviour (CI as colour bars) in session 3 for participants who have reached high R_2_ confidence (i.e., when 60 ≤ R ≤ 100), either gradually (i.e., R_0_ is not high, and R_0_ < R_1_ < R_2_) or persistently (i.e., participants remain highly confident from the beginning). Participants’ reactions are further classified by the C_3_ they saw (relative to R_2_) (see symbols on top). One participant (participant #32) who did not change his/her confidence for all 3 sessions in all questions (i.e., with mean of CI and S.D. of CI = 0, see S6 Fig) was excluded from analysis to better capture the behaviour of responsive participants.

If our psychological biases tend to push us to a higher confidence level and a high internal confidence tend to reduce our likelihood to conform to an opposite-sided individual (see above, also Ref. 26), are our biases leading us to a road of no return? Are we still open to modifying our beliefs when our confidence reached a certain high level? To address this question, we selected participants who has attained a high R_2_ (i.e., 60 ≤ R_2_ ≤ 100) either persistently (i.e., participant’s confidence was high from the beginning) or gradually (i.e., participant’s confidence was built gradually to a high level), and look into their CIs in the last confederation session (Fig 7). Interestingly, while the majority of participants (> 75%) in the persistent group remained indifferent to confederate challenges, a variety of conforming behaviours was detected in the gradual group. In particular for this latter group, participants who viewed a low C, regardless of the side, were more likely to anti-conform whereas under-/over-conformity was the dominant behaviour when C is high. In summary, one appears to retain a higher flexibility to conform when one’s confidence is constructed from others’.

## Discussion

Recent years have seen an immense growth in public video sharing and social video marketing. Many of these videos capture some forms of human motion, creating collective memory of various human activities. However, individual’s memory and even that of the crowd could be distorted during group communications. In the current study, we aim at examining the role of our and other’s confidence on memory conformity by asking how one’s confidence on his/her visual motion memory may be influenced by that of others’. Conformity was successfully reproduced. By tracking participant’s confidence transformation over three confederate sessions, our result suggested that conformity may not be purely an all-or-none behaviour but represents a spectrum of confidence change. Interestingly, participants show a bias toward accepting same-sided confederates holding similar confidence to theirs. This bias may explain the gradual increase in confidence and in turn a decrease in conforming tendency over confederate sessions, hence possibly promoting polarization.

### Experimental design

The current study is the first attempt to use confidence as a quantitative indicator of conformity level. In past studies, conforming tendency was usually reflected by the proportion of participants who complied to an opposite standpoint in a 2-AFC task involving memory recall [4]. However, we reckoned that participants may show confidence fluctuations in response to a confederate challenge without ever committing to a shift in sides, or do it only very lately. These fluctuations, although not as drastic, may still exert influence back on other members in a group and may accumulate in effect over time. Thus, we adopted here a more general definition of conformity: the tendency to match one’s confidence to others’ regardless whether there is side-shifting. We presented participants not only with opposite-sided answers but also with same-sided ones of varying strengths in order to map out confederate’s effect over a broad confidence space (Figs 3A, 4A and 4B). The conformity index was introduced (CI, see Materials and methods) for comparing more fairly the effect of confederate’s confidence on participant’s confidence across participants and sessions. With such, we were able to decompose conformity into anti-, no, under-, perfect and over-conforming behaviours, providing a more holistic picture of conforming pattern. This classification has further led us to discover a number of preferences that people displayed when engaging with confederates (discussed below).

### Side- and confidence-based biases during confidence conformity

Our major finding is that participants are most effectively influenced by confederates who are most similar to them in terms of both their side and memory confidence. This conclusion is reflected in a number of places: (1) participants tend to react to same-sided, similarly confident confederates by increasing their own confidence, even when the confederate is slightly less confident than themselves; (2) when the confederate is also same sided, but much more confident than the participant, the increase in confidence tend to be proportionately smaller; (3) when a same sided confederate is much less confident, participants also tend to have a smaller confidence increment, or even a confidence drop, comparing to those who encountered a similarly confident confederate; (4) when engaging with an opposite-sided confederates, participants experience a smaller confidence drop compared to the confidence enhancement brought by same-sided confederates; and (5) confederates tend to have a greater overall across-session influence on participant’s confidence change when their confidence is more similar to, rather than much higher or lower than the participant’s at each consecutive session. All in all, participants appeared to determine their degree of conformity by comparing confederates’ confidence with their own.

The above biases we observed during memory conformity reminds us of similar biases found in classical information processing and decision-making experiments. Specifically, prior beliefs have been reported to change how sensitive people are in perceiving information as well as to trigger confirmation bias, that is their reluctance in accepting new incompatible information [22, 23]. In certain ways, our memory test follows an analogous procedure of taking in information (i.e., confederate ratings) and making judgments (i.e., degree of conformity) as in earlier studies. Nonetheless, there are two fundamental distinctions:

First, the initial beliefs of our participants were constructed based on their memory, whereas past research relied either on pre-existing beliefs formed with undefined bases (e.g., position on gun control issue [24]) or on sensory assessments (e.g., determining the direction of dot movement [25]). In this latter case, it is difficult to tell whether confirmation bias arose from the act of making the assessment itself or actually from the memory of making such an assessment. Thus, we have shown more directly and definitively here that confirmation bias could be grounded on internal memory, no matter it is accurate or false memory.

Second, even when confidence ratings on participant’s decision were collected with a 2-AFC test in earlier studies on decision making, this rating was done subsequent to the binary decision [26, 27]. Thus, the reported confidence level may be itself the subject of post-decisional confirmation bias if it were regarded by the participants as a form of justification to their initial answers. In our current study, we have instead eliminated the 2-AFC step and informed the participants that scoring would be based solely on their confidence ratings. Hence, participants had to make their decision over a much larger number of choices and their self-reported confidence values most probably represent their memory strengths closer to the moment of decision-making. With such a refined rating scheme, we were able to see that confirmation bias may actually be prevalent across all participant’s confidence level. In fact, such confirmation bias appears no weaker as participant’s confidence drops; what actually changes is the confidence range that is being biased toward, which shifts lower when the initial confidence is low (Fig 3A). Hence, this suggested that the bias may be based on a reference point set at the self confidence level. This confidence bias exists in addition to the side bias.

The above observations provide new thoughts on how confirmation biases may be established. For instance, is there a hierarchy for side bias and confidence bias? Based on Fig 3A, participants seemed to regard side to be a more important feature so that they identified a same-sided but less confident confederate as a supporter by increasing their own confidence. A denser heat-map would be needed to further clarify participants’ conforming behaviours in dilemmas involving the two biases. An equally interesting question concerns the setup of reference points: in later sessions, would people compare the current confederate’s rating to their initial confidence (R_0_) or their revised confidence in the previous session (R_pre_)? Certain hints can be obtained from Fig 5: when two confederate confidence values are located equally away from R_0_ but in separate confederate trends (e.g., comparing C_2_ in Fig 5Axiii to 5C_1_ in Fig 5Axiv), they appear to exert different effects on participant’s confidence, suggesting that R_pre_, comparing to R_0_, is more likely to be the reference point. Future studies can test this hypothesis not only with uniformly increasing/decreasing trends of confederate ratings, but also with other trends (e.g., V-shape). Lastly, although we have successfully demonstrated conformity in our memory test, it remains an interesting and important question as to whether this change in confidence could be internalized and would indeed result in memory modification.

### Confidence polarization

In a group sharing some collective memory of an event, the aforementioned confirmation biases may strengthen the confidence of each member over time, thus potentially seed the development of mutual enhancement [28, 29]. If it is true that a higher self-confidence correlates with a lower conforming tendency, such enhanced confidence formed from mutual enhancement may evolve into an alarming scenario of polarization stabilized by feedforward loop, particularly in a group with low but similar level of confidence. Indeed, we have observed confidence polarization over as few as 3 confederate sessions (Fig 6). This may partly explain why human communities could become polarized on issues with certain motion memory components, such as whether violence has been overused by law enforcers, whether a public figure has misbehaved, etc. While the dividing situation may appear rather grim, our research has suggested a possible way of breaking out from the vicious cycle. We have found that people who build up their confidence upon others may retain a higher level of flexibility to change than those who have strong initial confidence. Thus, exposing oneself to contrasting information may be a starting point for a more balanced worldview. Future research will be needed to understand confidence polarization in more real-life situation.

## Materials and methods

### Participants

This research was approved by the University of Hong Kong Human Research Ethics Committee. A total of 38 participants (criteria: age 18–80 and able to read English) were recruited from the public through online invitation (mass email and WhatsApp messages) between March to June 2019. Experiments took place in the computer lab at the University of Hong Kong. All participants completed a same set of 90 randomized questions, giving altogether 3420 answers/datapoints (38 participants x 90 questions) for statistical power. No participants had been excluded in the process. All participants were informed of their right to discontinue participation at any time and have given consent to data collection; their responses were recorded digitally on a Google form (with digital signature and consent button). Personal information, including age, biological gender, ethnicity and education level, had also been collected (S3 Table). Our sample can be considered representative of a larger population.

To avoid suspicion, participants were told that the aim of the experiment was to investigate the effectiveness of human non-verbal communication and they were matched to a group with 3 other participants as well as randomly assigned an order to answer. Instructions on the memory test (including scoring scheme) and a short practice were given. Participants were motivated by being told that a cash reward (HKD$500) would be given to each participant in the best performing group after the whole experiment. In fact, the participant was the only human player in the group and was pre-set to be the fourth respondent (see below for further details). The cash reward was given to the best performer after the whole experiment instead. The whole experiment was about 30–40 minutes long. At the end, participants were debriefed and revealed the real aim of this study, i.e., to understand how one’s confidence would be transformed by other people’s when recalling visual memory of human motion.

### Memory test and confidence measurement

The experiment was created and conducted with PsychoPy v3.1.2 for MacOS (https://www.psychopy.org/index.html).

The experiment consists of three parts: (1) a study phase (<4 mins) in which participants were required to memorise computer-rendered human motions videos (45 in total, randomly ordered with a 1-s fixation frame in between) that would only be played once; (2) simple arithmetic tasks consisting of 10 simple addition and subtraction questions to prevent memory rehearsal; (3) a memory test consisting of 90 questions (45 targets and 45 lures) in each of which participants were required to indicate whether a certain motion has appeared before in the study phase. In this test phase, each motion video would be played only once after a 1-s fixation prompt. This was followed by 4 sub-sessions: the collection of an initial response and 3 successive confederate sessions. In all these sub-sessions, participants were required to answer the same question “Has the video appeared before?” with a slider under no time limit. The right and left end of the slider represents an absolute YES and NO, respectively, and participants were instructed to stop at any point in between (i.e., ranging from 100 to −100) to reflect their confidence toward the accuracy of their answers. A short training session has also been included. All participants have confirmed their understanding of the procedures before the test. In confederate sessions, participants were shown the rating of a confederate at the same time as the question was displayed.

To prevent participants from intentionally avoiding conforming actions, participants were deceived at the beginning of the experiment by being told that they would be randomly assigned to a group of four with three other groupmates and their common goal would be to achieve the highest group score among all competing groups. Score for each subquestion would be calculated as follows: score = + slider value (if answer is correct) and score = − slider value (if answer is wrong). The scores will not be displayed on the screen during the test, and participants had been informed about these rules. Participants were also told that their adjustments will be recorded and counted toward their final score. If they were the first member of the group, they only had to indicate their initial answers. Else, the answer(s) of the previous group member(s) would be shown, and they would have extra chance(s) to answer to the same question. In other words, if they were the fourth person of the group, they would have three extra rounds to earn points in addition to the initial round. All participants had indicated an understanding and consent to the above procedures. However, in reality, the whole process was not random, the group members’ answers were computer-generated (see below for further details), and the participants were all assigned to be the fourth member. All 4 responses in a question were tracked and saved (38 participants x 90 questions = 3420 answers).

In order to familiarize the participants with the experiment and function keys, a short training session was given to the participants after the demographic questionnaire at the beginning of the test. In this training, 3 motion videos were shown. Participants were told to be the second member of the group and were guided to complete the initial questions and the subsequent confederate sessions. All questions followed the format of the real test.

### Motion videos

All the motion videos were recorded using a Window 10 computer. Motions were captured with a motion sensor, Xbox One Kinect 2, which supports the detection of human motion and gestures. NI Mate (Version 2.14) and Kinect Studio (Version 2.0.1410.19000) was used to transform the motions of the real-person actor to black and white skeletal animations, which were screen recorded into 1280 px × 720 px videos (see Fig 1). A total of 148 videos were recorded, each containing one motion and lasts for 3 sec. Fifty-five videos with abnormal shaking or joint linkage were later screened out, leaving 93 in total for use. First, we classified the recorded motion video based on the body part involved into arm(s), leg(s), or complex motion (S1 Table, columns 3–5). Four raters were then recruited to watch each video, interpret the motions’ meaning and to provide feedbacks on the similarities of the actor’s gestures. Motions with similar meanings and/or gestures were assigned into the same group, giving 21 groups and 47 solitary motions. Within each group, motions were divided into targets (those appeared in both the study and the test phase) and lures (those appeared only in the test phase) (S1 Table, column 6). Ungrouped motions were assigned randomly as targets or lures under the principle of having the same number of motions involving particular body part(s) in the two groups. The three remaining motions (2 x arm motion, and 1 x complex motion) were used in the practice session before the study phase in order to prepare participants for the experiment. The list of motions was summarized in S1 Table.

### Confederate conditions

We divided the response space into 6 ranges: (+60 to +100), (+30 to +59), (0 to +29), (−1 to −29), (−30 to −59) and (−60 to −100). If a participant’s initial response (R_0_) lied within a designated range, he/she had a certain pre-defined probability of encountering a set of three confederate answers out of 5 sets of conditions. In the first 3 conditions, the three confederate answers of a single test showed trends with different ascending/descending rates. In the 4th condition, the three confederate answers were set to be constant. The last condition had no constraint. A random value within ±10 was sampled from a uniform distribution and added to condition 1–4 as noise to reduce conspicuity. The conditions randomly appeared with preset probability. The list of conditions was summarized in S2 Table.

### Data analyses

Data analyses were done by using R studio (version 3.6.0 for macOS High Sierra 10.13.6). Graphs were produced by using Plot.ly (version 4.9.2), ggplot (version 3.3.0) and Heatmaply (version 3.6.2).

In the current study, we introduced the conformity index (CI) as a measure of participant’s level of conformity. In general terms, CI represents the degree of change in participant’s response per unit difference between the confederate’s and participant’s confidence. Mathematically,

$$\text{Conformity}\;\text{Index}(\text{CI})=\frac{{\text{R}}_{\text{post}}-{\text{R}}_{\text{pre}}}{{\text{C}-\text{R}}_{\text{pre}}}$$ (2)

Different ranges of CI correspond to different conformity behaviour: anti-conformity (CI < 0), no conformity (CI = 0), under-conformity (0 < CI < 1), perfect conformity (CI = 1) and over-conformity (CI > 1). It should be noted that CI is invalid when C = R_pre_, and data points with invalid CI were excluded from all data analyses involving CI.

Subsets of data were selected or excluded to address particular research questions. Specifically, in Fig 2 and S3 Fig, only data with a confederate value C_1_ on the opposite side to the initial response (R_0_) were included. In Figs 3A and 4 (left panels), data points with invalid CI (i.e., C = R_pre_) were excluded. In Fig 5, only data with either an upward (C_1_ < C_2_ < C_3_) or a downward trend (C_1_ > C_2_ > C_3_) of confederate ratings over the three confederate sessions were included. Trials with uneven steepness of slopes (i.e., when the difference between the slope of C_1_-C_2_ and the slope of C_2_-C_3_ exceeds 20) were eliminated. In S6 Fig, participant #32 was eliminated as no confidence adjustment is observed throughout the whole test, suggesting that he/she might disregard confederates in all occasions.

As trials with an answer “Yes” and those with “No” seem to show quite symmetric conforming behavioural patterns in Figs 3A, 4A and 4B (Left), data with R_0_< 0 was reflected over the origin and combined with data with in R_0_≥ 0 Figs 3C, 5A and 6 and S5 Fig.

For easier comparison, in Fig 5, we calculated the rate of participant’s confidence change across the three confederate sessions by dividing the total change in confidence by the slope of C_1_-C_2_-C_3_ with the following equation:

$$\text{Rate}\;\text{of}\;\text{change}=\frac{{\text{R}}_{\text{3}}-{\text{R}}_{\text{0}}}{\left(\frac{{(\text{C}}_{\text{2}}-{\text{C}}_{\text{1}}{)+(\text{C}}_{\text{3}}{-\text{C}}_{\text{2}})}{\text{2}}\right)}$$ (3)

For better visualization, subject-video in Fig 6 was clustered with respect to its trend of participant’s response over session and was sorted in descending order of (R_0_ + R_1_ + R_2_ + R_3_). S5 Fig followed the same order as in Fig 6A.

## Supporting information

S1 Fig Variation of participants’ initial confidence level. Participants’ average initial confidence (R_0_) level (mean ± SD) across target videos (blue) in descending order and lure videos (red) in ascending order. Means for target range from +45.2 to -7.8. Means for lures range from -53.8 to +15.3.
(PDF)

S2 Fig Effect of prior experience on current response. Each box shows the Pearson correlation coefficient between (C − R_pre_) and (R_post_− R_pre_) ordered according to video number in the test phase. Coefficients are means of all participants (n = 38 participants).
(PDF)

S3 Fig Different conforming behaviours to an opposite-sided confederate in confederate sessions 2 and 3. Same as Fig 2 but for session 2 (panel A, n = 370) and session 3 (panel B, n = 297).
(PDF)

S4 Fig Conforming behaviours with respect to R_pre_ and confederate confidence in sessions 2 and 3. Same as Fig 3B but for session 2 (panel A, n = 3376) and session 3 (panel B, n = 3365).
(PDF)

S5 Fig Heat-map plot of confederate values across sessions. Each line shows the confederate values of the three sessions presented to a participant in a particular video question (n = 38 participants x 90 questions = 3420 lines). Lines are arranged in the same order as in Fig 6 to show that the gradual increase in confidence seen in Fig 6 is not purely due to a gradual increase in confederates’ confidence across sessions.
(PDF)

S6 Fig Participants’ mean and S.D. of CI in the 3 sessions. Each dot shows the averaged mean and S.D. of all the CI’s for all 90 questions of a particular participant in the indicated session (n = 38). Participant’s numbers are marked next to the point. An adamant participant (red) with zero mean and S.D. of CI was excluded from the analysis in Fig 7.
(PDF)

S1 Table List of target and lure motion videos. (PDF)

S2 Table Equations for generating confederate values. A set of three confederate values (C_1_, C_2_ and C_3_) for 5 different conditions were generated for each of the following ranges of initial response (R_0_) based on the following equations. x1, x2 and x3 were noise within ±10 randomly generated by computer and added for reducing conspicuity felt by the participants. A particular condition was then randomly chosen by computer based on the participant’s R_0_ to a particular test video with the probabilities listed below.
(PDF)

S3 Table Demographic information. Total number of participants = 38.
(PDF)
