# Supplementary material for: Side- and similarity-biases during confidence conformity
Source: PLoS One. 2021 Jul 16;16(7):e0253577. doi: 10.1371/journal.pone.0253577 (PMC8284640; doi:10.1371/journal.pone.0253577)
Supplement: S1 Fig — Participants’ average initial confidence (R0) level (mean ± SD) across target videos (blue) in descending order and lure videos (red) in ascending order. Means for target range from +45.2 to -7.8. Means for lures range from -53.8 to +15.3. (PDF) [file pone.0253577.s001.pdf]

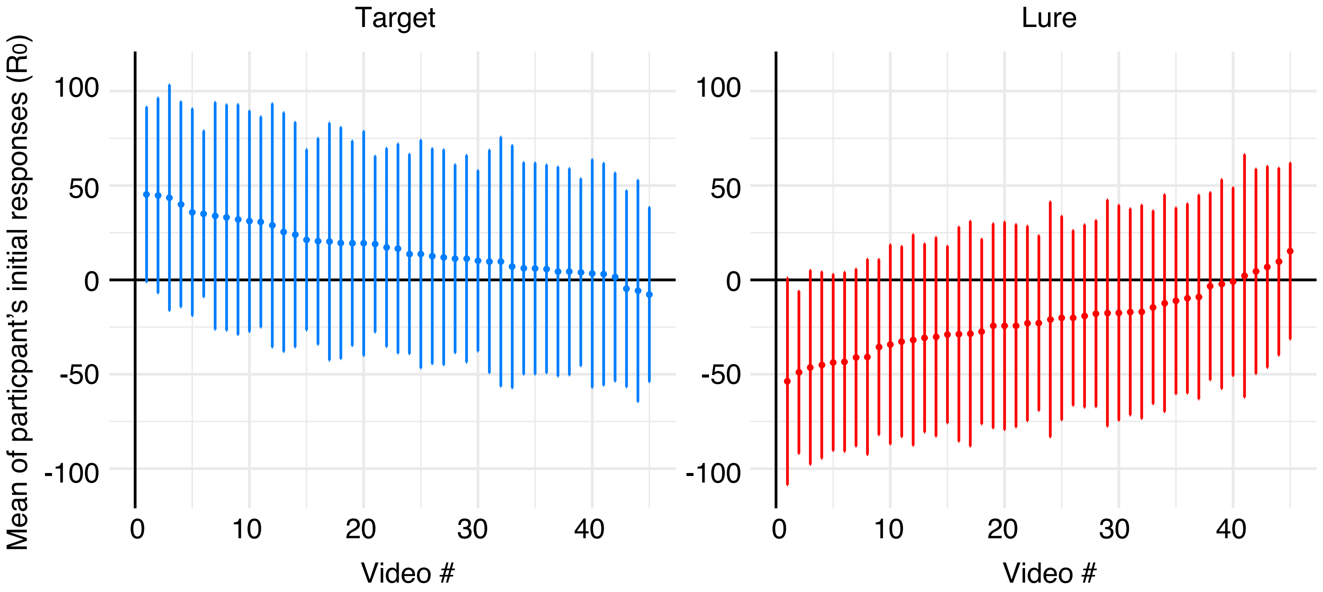

S1 Fig. Variation of participants' initial confidence level. Participants' average initial confidence (R_0) level (mean ± SD) across target videos (blue) in descending order and lure videos (red) in ascending order. Means for target range from +45.2 to -7.8. Means for lures range from -53.8 to +15.3.
