# Supplementary material for: Side- and similarity-biases during confidence conformity
Source: PLoS One. 2021 Jul 16;16(7):e0253577. doi: 10.1371/journal.pone.0253577 (PMC8284640; doi:10.1371/journal.pone.0253577)
Supplement: S2 Fig — Each box shows the Pearson correlation coefficient between (C − Rpre) and (Rpost− Rpre) ordered according to video number in the test phase. Coefficients are means of all participants (n = 38 participants). (PDF) [file pone.0253577.s002.pdf]

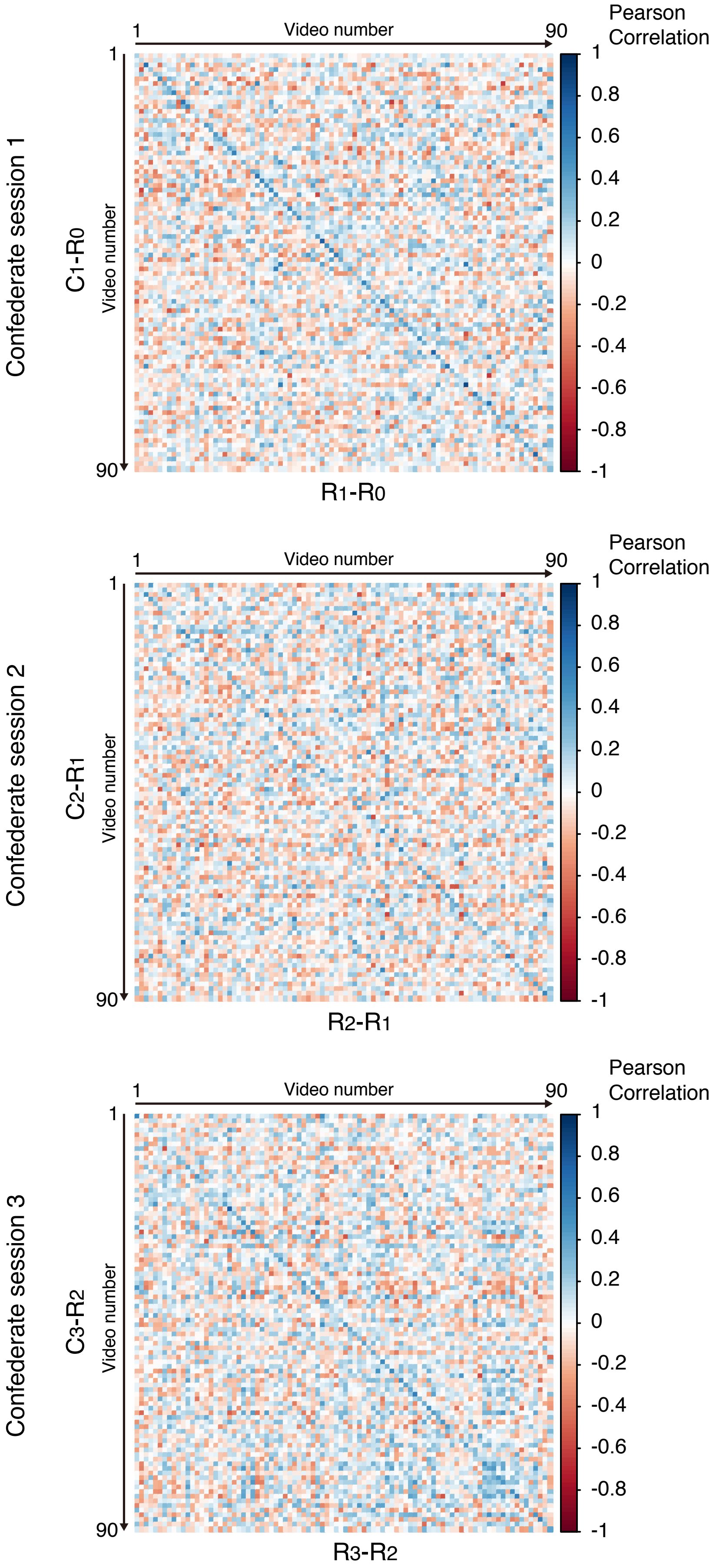

S2 Fig. Effect of prior experience on current response. Each box shows the Pearson correlation coefficient between ($C - R_{pre}$) and ($R_{post} - R_{pre}$) ordered according to video number in the test phase. Coefficients are means of all participants (n = 38 participants).
