# Supplementary material for: Side- and similarity-biases during confidence conformity
Source: PLoS One. 2021 Jul 16;16(7):e0253577. doi: 10.1371/journal.pone.0253577 (PMC8284640; doi:10.1371/journal.pone.0253577)
Supplement: S3 Fig — Same as Fig 2 but for session 2 (panel A, n = 370) and session 3 (panel B, n = 297). (PDF) [file pone.0253577.s003.pdf]

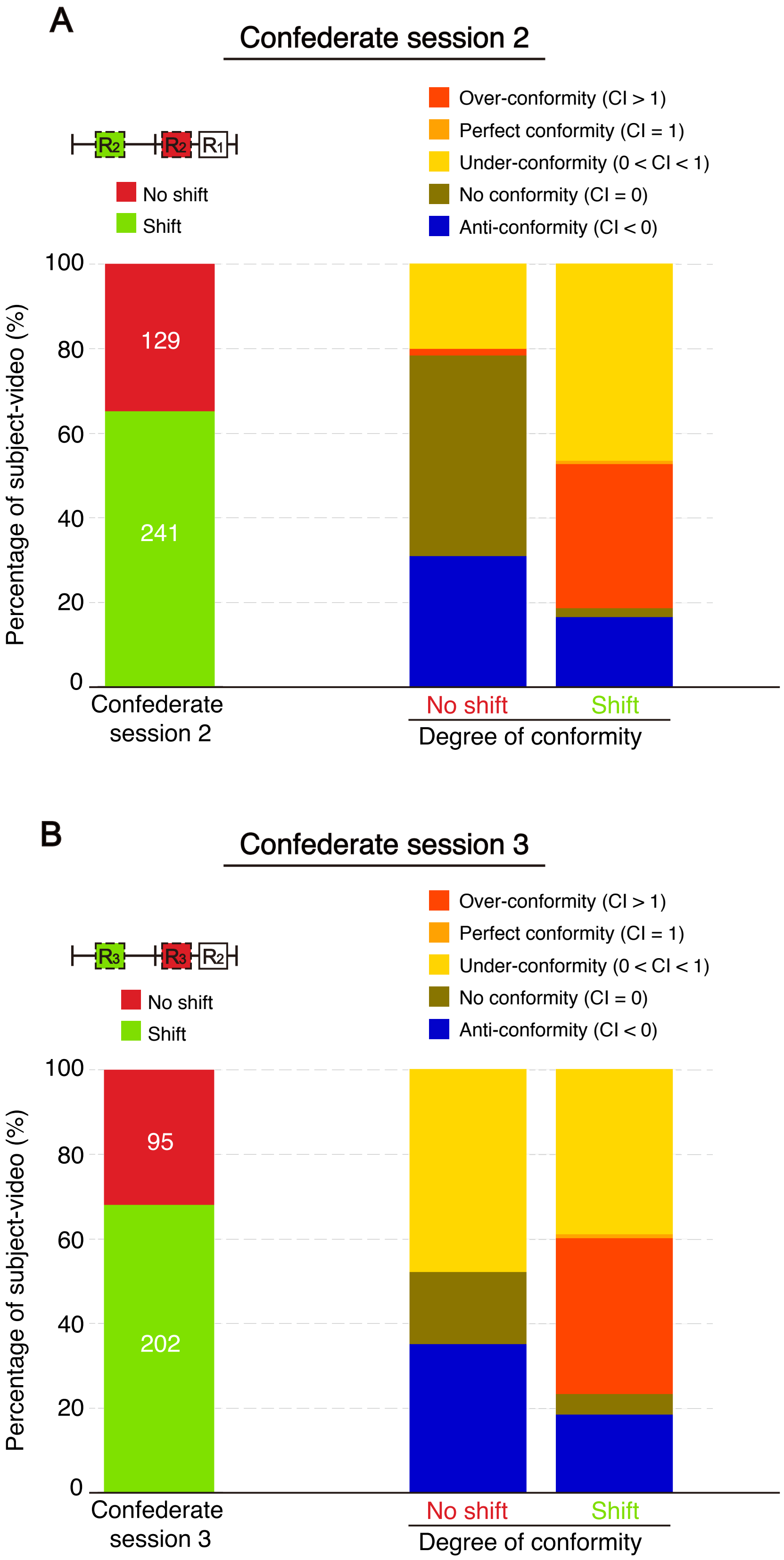

S3 Fig. Different conforming behaviours to an opposite-sided confederate in confederate sessions 2 and 3. Same as Fig 2 but for session 2 (panel A, n = 370) and session 3 (panel B, n = 297).
