# Supplementary material for: Side- and similarity-biases during confidence conformity
Source: PLoS One. 2021 Jul 16;16(7):e0253577. doi: 10.1371/journal.pone.0253577 (PMC8284640; doi:10.1371/journal.pone.0253577)
Supplement: S4 Fig — Same as Fig 3B but for session 2 (panel A, n = 3376) and session 3 (panel B, n = 3365). (PDF) [file pone.0253577.s004.pdf]

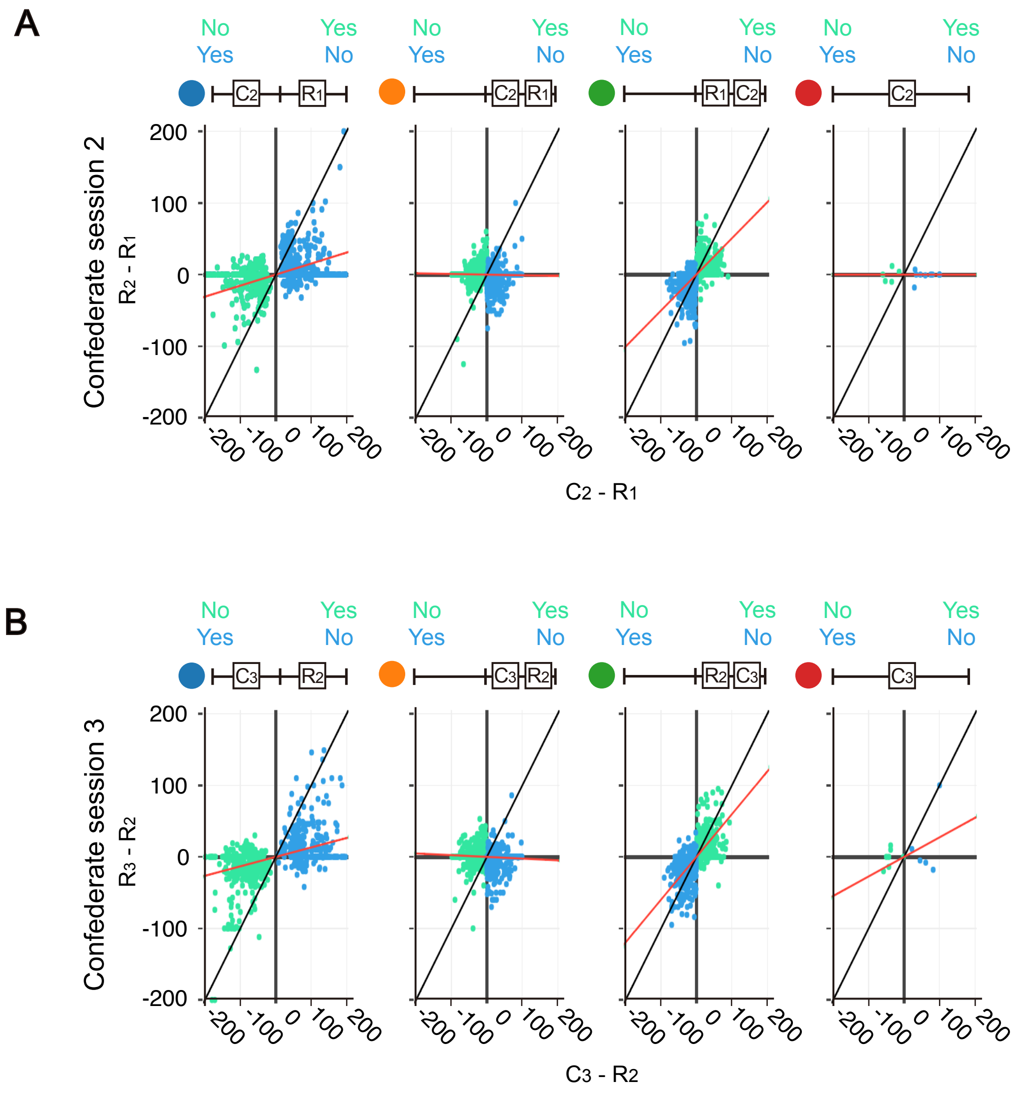

S4 Fig. Conforming behaviours with respect to R_{pre} and confederate confidence in sessions 2 and 3. Same as Fig 3B but for session 2 (panel A, n = 3376) and session 3 (panel B, n = 3365).
