# Supplementary material for: Side- and similarity-biases during confidence conformity
Source: PLoS One. 2021 Jul 16;16(7):e0253577. doi: 10.1371/journal.pone.0253577 (PMC8284640; doi:10.1371/journal.pone.0253577)
Supplement: S5 Fig — Each line shows the confederate values of the three sessions presented to a participant in a particular video question (n = 38 participants x 90 questions = 3420 lines). Lines are arranged in the same order as in Fig 6 to show that the gradual increase in confidence seen in Fig 6 is not purely due to a gradual increase in confederates’ confidence across sessions. (PDF) [file pone.0253577.s005.pdf]

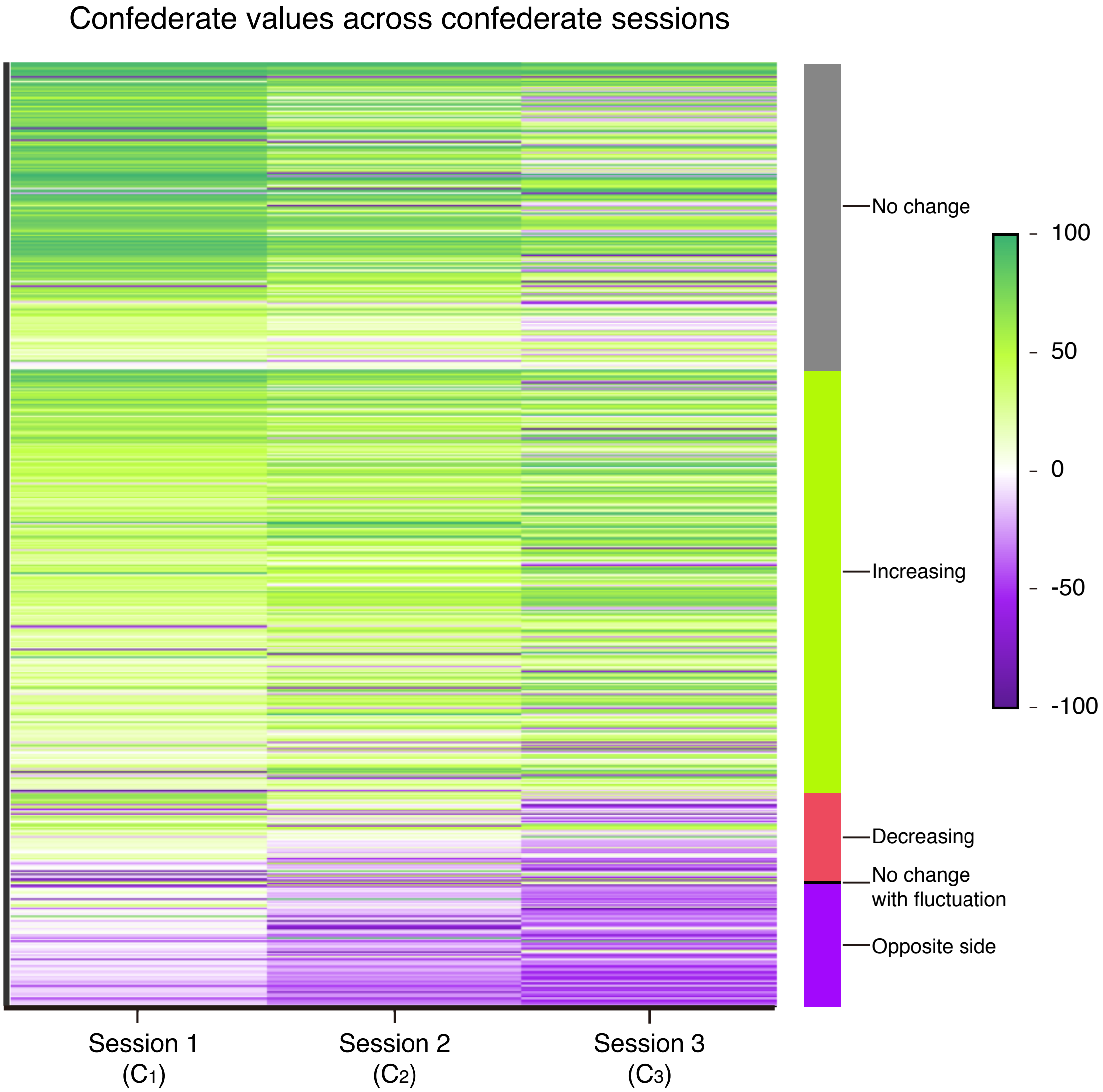

S5 Fig. Heat-map plot of confederate values across sessions. Each line shows the confederate values of the three sessions presented to a participant in a particular video question (n = 38 participants x 90 questions = 3420 lines). Lines are arranged in the same order as in Fig 6 to show that the gradual increase in confidence seen in Fig 6 is not purely due to a gradual increase in confederates' confidence across sessions.
