# Supplementary material for: Side- and similarity-biases during confidence conformity
Source: PLoS One. 2021 Jul 16;16(7):e0253577. doi: 10.1371/journal.pone.0253577 (PMC8284640; doi:10.1371/journal.pone.0253577)
Supplement: S6 Fig — Each dot shows the averaged mean and S.D. of all the CI’s for all 90 questions of a particular participant in the indicated session (n = 38). Participant’s numbers are marked next to the point. An adamant participant (red) with zero mean and S.D. of CI was excluded from the analysis in Fig 7. (PDF) [file pone.0253577.s006.pdf]

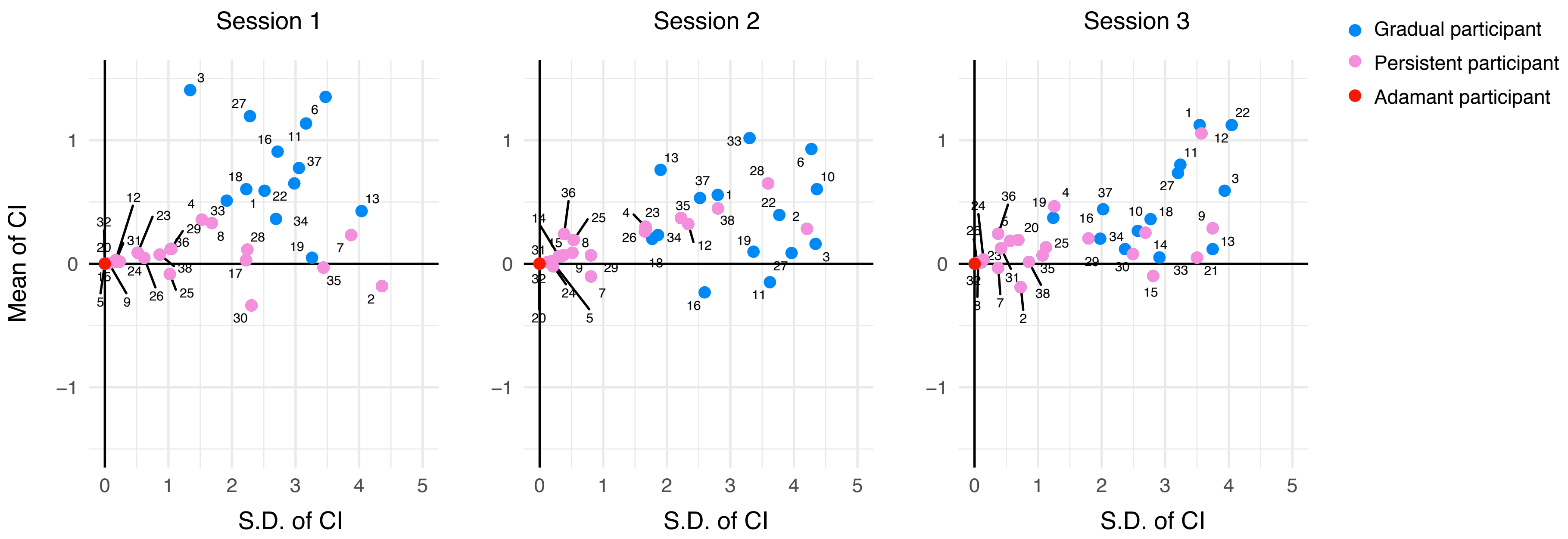

S6 Fig. Participants' mean and S.D. of CI in the 3 sessions. Each dot shows the averaged mean and S.D. of all the CI's for all 90 questions of a particular participant in the indicated session (n = 38). Participant's numbers are marked next to the point. An adamant participant (red) with zero mean and S.D. of CI was excluded from the analysis in Fig 7.
