# Supplementary material for: Side- and similarity-biases during confidence conformity
Source: PLoS One. 2021 Jul 16;16(7):e0253577. doi: 10.1371/journal.pone.0253577 (PMC8284640; doi:10.1371/journal.pone.0253577)
Supplement: S1 Table — (PDF) [file pone.0253577.s007.pdf]

S1 Table. List of target and lure motion videos

Target videos

no.	Video Name	Arm(s)	Leg(s)	Complex	Group
1	"Threatening"	■			
2	Sitting down			■	
3	Towing head	■			
4	Bending towards left			■	7
5	Crossing steps		■		
6	Shaking right leg slowly		■		4
7	Cleaning windows	■			
8	Jumping		■		3
9	Pulling	■			21
10	Stumbling forward			■	8
11	Hands up obliquely			■	
12	Bouncing the ball			■	
13	Kneeling down and pray			■	5
14	Music-conducting	■			14
15	Folding arms	■			
16	Star-shaped jumping			■	3
17	"Kung-fu"			■	
18	"Old man"			■	
19	Yoga			■	9
20	Squatting			■	
21	Stirring with one hand	■			
22	"Bird flying"	■			2
23	Pushing away with one hand	■			13
24	Standing in star-shaped			■	20
25	Clapping hand			■	15
26	Drawing circles with both hands	■			1
27	Marching			■	17
28	Shaking both hands	■			6
29	Face Palm	■			19
30	Extending empty hands	■			
31	Rubbing hair	■			12
32	Surrendering with both hands	■			
33	Weightlifting			■	
34	Pounding the table	■			
35	"Go away"	■			18
36	Hands over shoulder	■			
37	Kicking			■	16
38	Dancing			■	11
39	Akimboing	■			
40	"The Thinker"			■	
41	Lifting up a box			■	
42	Handshaking with other			■	
43	Balancing			■	
44	Ramming			■	10
45	Pushing			■	

Lure videos

no.	Video Name	Arm(s)	Leg(s)	Complex	Group
1	"Teapot"			■	7
2	Crawling			■	
3	"Kung-fu"			■	
4	Running			■	17
5	Dancing			■	
6	"Kung-fu"			■	
7	Stumbling backward			■	8
8	Rotating hand	■			1
9	Dancing			■	11
10	Drawing circles with both hands	■			1
11	Turning waist			■	
12	Bending			■	
13	Dodging	■			10
14	"Butterfly flying"	■			20
15	Shooting basketball	■			
16	Rocking feet		■		
17	Rope skipping			■	
18	Crouching down and put down the carring box			■	
19	Washing hair	■			12
20	Skiing			■	
21	Bowling			■	
22	"Flying"			■	2
23	Kneeling down and stand up			■	5
24	Punching			■	
25	Crossing legs	■			16
26	"Smelly"	■			18
27	Dancing ballet			■	9
28	Blocking	■			19
29	Sand-hammering	■			6
30	Wave-liked hand motion	■			
31	Crossing the threshold			■	4
32	Discoing	■			
33	Catching			■	
34	Raising up right foot		■		
35	Washing hands			■	15
36	Hands tilted upwards (worshipping)			■	
37	Slapping	■			21
38	Seeing things with a distance	■			
39	Crouching down		■		5
40	"Bird flying "	■			2
41	Jumping (2)			■	3
42	Frying food with a pan	■			14
43	Saluting	■			
44	Waving	■			
45	"Come over"	■			13

Videos in the training session

no.	Video Name
1	Raising one hand
2	Pulling
3	Playing volleyball
