# Supplementary material for: Side- and similarity-biases during confidence conformity
Source: PLoS One. 2021 Jul 16;16(7):e0253577. doi: 10.1371/journal.pone.0253577 (PMC8284640; doi:10.1371/journal.pone.0253577)
Supplement: S2 Table — A set of three confederate values (C1, C2 and C3) for 5 different conditions were generated for each of the following ranges of initial response (R0) based on the following equations. x1, x2 and x3 were noise within ±10 randomly generated by computer and added for reducing conspicuity felt by the participants. A particular condition was then randomly chosen by computer based on the participant’s R0 to a particular test video with the probabilities listed below. (PDF) [file pone.0253577.s008.pdf]

**S2 Table. Equations for generating confederate values**

A set of three confederate values ($C_1$, $C_2$ and $C_3$) for 5 different conditions were generated for each of the following ranges of initial response ($R_0$) based on the following equations. $x_1$, $x_2$ and $x_3$ were noise within ±10 randomly generated by computer and added for reducing conspicuity felt by the participants. A particular condition was then randomly chosen by computer based on the participant's $R_0$ to a particular test video with the probabilities listed below.

| Range of $R_0$ | Condition | $C_1$ | $C_2$ | $C_3$ | Probability |
|---|---|---|---|---|---|
| +60 to +100 | 1 | $R_0 - 10 + x_1$ | $C1 - 10 + x_2$ | $C2 - 10 + x_3$ | 25% |
| | 2 | $R_0 - 20 + x_1$ | $C1 - 20 + x_2$ | $C2 - 20 + x_3$ | 25% |
| | 3 | $R_0 - 40 + x_1$ | $C1 - 40 + x_2$ | $C2 - 40 + x_3$ | 25% |
| | 4 | $R_0 + x_1$ | $R_0 + x_2$ | $R_0 + x_3$ | 12.5% |
| | 5 | Random | Random | Random | 12.5% |
| +30 to +59 | 1 | $R_0 + 10 + x_1$ | $C1 + 10 + x_2$ | $C2 + 10 + x_3$ | 25% |
| | 2 | $R_0 - 20 + x_1$ | $C1 - 20 + x_2$ | $C2 - 20 + x_3$ | 25% |
| | 3 | $R_0 - 10 + x_1$ | $C1 - 10 + x_2$ | $C2 - 10 + x_3$ | 25% |
| | 4 | $R_0 + x_1$ | $R_0 + x_2$ | $R_0 + x_3$ | 12.5% |
| | 5 | Random | Random | Random | 12.5% |
| 0 to +29 | 1 | $R_0 + 10 + x_1$ | $C1 + 10 + x_2$ | $C2 + 10 + x_3$ | 25% |
| | 2 | $R_0 + 20 + x_1$ | $C1 + 20 + x_2$ | $C2 + 20 + x_3$ | 25% |
| | 3 | $R_0 - 20 + x_1$ | $C1 - 20 + x_2$ | $C2 - 20 + x_3$ | 25% |
| | 4 | $R_0 + x_1$ | $R_0 + x_2$ | $R_0 + x_3$ | 12.5% |
| | 5 | Random | Random | Random | 12.5% |
| –1 to –29 | 1 | $R_0 - 10 + x_1$ | $C1 - 10 + x_2$ | $C2 - 10 + x_3$ | 25% |
| | 2 | $R_0 - 20 + x_1$ | $C1 - 20 + x_2$ | $C2 - 20 + x_3$ | 25% |
| | 3 | $R_0 + 20 + x_1$ | $C1 + 20 + x_2$ | $C2 + 20 + x_3$ | 25% |
| | 4 | $R_0 + x_1$ | $R_0 + x_2$ | $R_0 + x_3$ | 12.5% |
| | 5 | Random | Random | Random | 12.5% |
| –30 to –59 | 1 | $R_0 - 10 + x_1$ | $C1 - 10 + x_2$ | $C2 - 10 + x_3$ | 25% |
| | 2 | $R_0 + 20 + x_1$ | $C1 + 20 + x_2$ | $C2 + 20 + x_3$ | 25% |
| | 3 | $R_0 + 10 + x_1$ | $C1 + 10 + x_2$ | $C2 + 10 + x_3$ | 25% |
| | 4 | $R_0 + x_1$ | $R_0 + x_2$ | $R_0 + x_3$ | 12.5% |
| | 5 | Random | Random | Random | 12.5% |
| –60 to –100 | 1 | $R_0 + 10 + x_1$ | $C1 + 10 + x_2$ | $C2 + 10 + x_3$ | 25% |
| | 2 | $R_0 + 20 + x_1$ | $C1 + 20 + x_2$ | $C2 + 20 + x_3$ | 25% |
| | 3 | $R_0 + 40 + x_1$ | $C1 + 40 + x_2$ | $C2 + 10 + x_3$ | 25% |
| | 4 | $R_0 + x_1$ | $R_0 + x_2$ | $R_0 + x_3$ | 12.5% |
| | 5 | Random | Random | Random | 12.5% |
