# Supplementary material for: Side- and similarity-biases during confidence conformity
Source: PLoS One. 2021 Jul 16;16(7):e0253577. doi: 10.1371/journal.pone.0253577 (PMC8284640; doi:10.1371/journal.pone.0253577)
Supplement: S3 Table — Total number of participants = 38. (PDF) [file pone.0253577.s009.pdf]

S3 Table. Demographic information.

Total number of participants = 38.

Gender	
**Women**	**Men**
26	12

Ethnicity	
**White**	**Asian**
1	37

Age					
**18-24**	**25-34**	**35-44**	**45-54**	**55-64**	**65-74**
17	13	2	3	2	1

Education				
**Lower than high school degree diploma**	**High school degree or equivalent**	**Bachelor degree**	**Master degree**	**Doctorate**
3	3	18	9	5
